# The Content of Minerals in the PCOS Group and the Correlation with the Parameters of Metabolism

**DOI:** 10.3390/nu13072214

**Published:** 2021-06-28

**Authors:** Kamila Pokorska-Niewiada, Agnieszka Brodowska, Małgorzata Szczuko

**Affiliations:** 1 Department of Toxicology, Dairy Technology and Food Storage, West Pomeranian University of Technology in Szczecin, 71-459 Szczecin, Poland; 2 Department of Gynecology, Endocrinology and Gynecological Oncology, Pomeranian Medical University in Szczecin, 71-252 Szczecin, Poland; agnieszka.brodowska@pum.edu.pl; 3 Department of Human Nutrition and Metabolomics, Pomeranian Medical University in Szczecin, 71-460 Szczecin, Poland

**Keywords:** polycystic ovary syndrome, microelements, erythrocytes, glycemic markers, lipids metabolism

## Abstract

Polycystic ovary syndrome (PCOS) is a common disease in women of childbearing age. It is characterized by excessive androgen production, ovulation disorders, and developing metabolic syndrome. The aim of the study was to check whether selected minerals were related to the pathophysiological mechanisms of PCOS. The concentrations of minerals were determined using an inductively coupled atomic plasma-emission spectrometer (ICP-AES Jobin Yvon JY-24). Blood samples from PCOS and control women were collected, processed, and digested with a microwave system in women with PCOS with and without insulin resistance and in the control group. It was found: zinc (Zn)-10.14 ± 2.11, 9.89 ± 1.44 and 10.30 ± 1.67; nickel (Ni) 0.001 ± 0.0009, 0.001 ± 0.0006 and 0.002 ± 0.00001; iron (Fe) 868.0 ± 155.8, 835.3 ± 156.4 and 833.0 ± 94.6; manganese (Mn) 0.017 ± 0.006, 0.017 ± 0.008 and 0.020 ± 0.009; copper (Cu) 0.714 ± 0.129, 0.713 ± 0.114 and 0.761 ± 0.146; magnesium (Mg) 48.4 ± 8.3, 50.0 ± 8.4 and 45.3 ± 10.7; sodium (Na) 374.3 ± 84.3, 396.3 ± 66.6 and 367.9 ± 88.9; potassium (K) 2541.8 ± 330.9, 2409.6 ± 347.1 and 2336.9 ± 211.4 (µg/g). Some micronutrient deficiencies may have a negative effect on the lipid profile in PCOS patients (Ni, Na). Further studies are needed to better understand dependencies.

## 1. Introduction

Polycystic ovary syndrome (PCOS) is a disease that, according to various researchers, occurs in 10–21% of women of childbearing age [1]. The syndrome was first defined by Stein and Leventhal in 1935 and is now one of the most common endocrine diseases, with clinical symptoms such as hyperandrogenism, menstrual disorders, infertility, and hirsutism [2,3]. PCOS is responsible for approximately 70% of cases of anovulatory infertility [4,5]. Recently, scientists have been increasingly paying attention to factors causing pituitary dysfunction, namely inflammatory processes of the body against the background of increased oxidative stress, lifestyle, diet, etc., which play a fundamental role in PCOS-related diseases and the accompanying metabolic syndromes [6].

There is ample evidence that minerals are important for female reproductive function. In particular, minerals can be associated with ovulation, metabolism, and hormone management. There are over 60 minerals in varying amounts in human cells. Each of them plays an important role in various processes in the human body [7]. Minerals are components of enzymes or coenzymes that control a wide range of energetic and metabolic reactions and act as compounds or coordinators of specific cellular functions in major body tissues [8]. The most important feature is their interaction [9]. So far, most studies on minerals and reproductive function, especially reproductive hormones, have been based on concentrations of minerals in human serum [10,11,12] or in animal models [13,14].

The level of elements in the serum informs us about the current state of the body, but does not tell about the reserves accumulated in blood cells. This is why we decided to study the levels of micro and macroelements in red blood cells. The use of vitamin and mineral complexes in the treatment of PCOS is justified in the modern scientific literature.

The role of macro and micronutrients in the occurrence of this disease is almost unknown. There are only individual studies devoted to studying the composition of macro- and microelements in women with PCOS. Many authors observe higher levels of Cu, Zn [15], Ca, and Mn [7] in women with PCOS. Chavarro el al [5], analyzing the relationship between the consumption of various forms of iron and ovulatory infertility, found that the use of iron supplements with high iron content was associated with a reduced risk of ovulatory infertility.

Copper, zinc, and manganese are the basic micronutrients that, along with numerous proteins and metalloenzymes, are essential in the metabolic systems of cells and in the oxidative stress pathways. In the first enzymatic step, superoxide dismutase (SOD) and glutathione-dependent enzymes (GSH) are the key antioxidants, copper-zinc SOD (Cu, Zn-SOD or SOD1) is found in the cytosol, and manganese SOD (Mn-SOD or SOD2) is located in the mitochondria [16,17,18,19].

Total-serum magnesium and calcium levels do not appear to change in infertile women. However, Grossi et al. [20] found a downward trend in total magnesium and calcium levels in relation to the increase in estrogens.

Magnesium is one of the cations found in the human body that takes part in energy transformations and determines the proper course of hormonal reactions and insulin secretion. The secretion of insulin is initiated by the influx of Ca^2+^, which is competitively inhibited by extracellular Mg^2+^. This may explain the inverse correlation between serum Mg^2 +^ concentration and serum-insulin concentration. Magnesium is closely related to type-2 diabetes and other metabolic diseases that accompany women with PCOS [21].

In conclusion, the exact cause of PCOS is still unknown, but micro and macroelements have been shown to be important in the pathogenesis and metabolism of PCOS. Abnormal mineral content may serve as an indicator of illness [22,23].

Overall, it is remarkable how little research has been done on this important issue for women with PCOS. Suspecting that the levels of trace elements in women with PCOS (which have not been adequately researched so far) may be related to oxidative stress and affect the course of their metabolism, in the present study, we decided to investigate the levels of some essential elements in erythrocytes and compare them with glycemic markers and lipids metabolism.

## 2. Materials and Methods

### 2.1. Participants

Forty-seven women (18–38 years old) diagnosed with PCOS participated in the study. The diagnosis was based on ultrasound (Ultrasound Voluson 730, GE, Baden, Switzerland) and the Rotterdam criteria (2003).

The characteristics of the respondents are presented in Table 1 and Table 2. The control group (CG) consisted of healthy women (*n* = 16) with a normal menstrual cycle, mean age 29 ± 4.4 years, height 1.68 ± 0.05 m, and normal BMI 23.3 ± 2.6 (m^2^/kg) who were not hospitalized.

Recognizing that dietary intake of minerals plays a significant role in body concentrations, the diet of PCOS patients and healthy patients was analyzed. Data on the diets of the studied women were compiled on the basis of food diaries recorded for 4 days (two weekdays and two weekend days, and a one-day nutritional record for the last 24 h). The content of selected trace elements in the diet was calculated using the Diet 4.0 nutritional program recommended by the National Food and Nutrition Institute. The diets did not differ statistically with regard to the content of trace elements; therefore, this aspect was omitted in the study (Table 3).

### 2.2. Methods of Measurement of Body Composition

For women, anthropometric measurements were performed according to the guidelines contained in the laboratory and field measurements of body composition [24]. Total body adiposity was measured under standardized conditions by tetra-polar bioelectrical impedance analysis (BIA-101, Akern, Florence, Italy). Total body water, extracellular water, intracellular water, fat mass, and muscle mass were measured. The anthropometric characteristics of the study group are presented in Table 1.

### 2.3. Blood Sample Collection

Fasting blood samples were taken from PCOS women and controls in EDTA (ethylenediaminetetraacetic acid) tubes. Elisa test was performed. Blood samples were immediately placed on ice/refrigerated and the samples were centrifuged (3500 rpm for 10 min at 4 °C) for 15 min after sampling. The red blood cell samples were immediately stored in Eppendorf tubes at −80 °C.

### 2.4. Biochemical Measurements

Biochemical analyses were performed in the University Hospital’s Laboratory, SPSK2 Szczecin, Poland. Glucose was analyzed by an enzymatic method using hexokinase (Cobas Integra 400 plus Roche, Switzerland). Insulin resistance (IR) was assessed using the fasting glucose/insulin ratio (GIR).

HOMA index was calculated as: concentration of the fasting insulin (μU/mL) × fasting glucose (mmol/L)/22.5.

The assessment of insulin resistance was based on the HOMA-IR ≥ 2.5. On this basis, two groups of patients were distinguished: PCOS-IR (PCOS with insulin resistance) and PCOS-NIR (PCOS without insulin resistance). Lipid profile was evaluated by the enzymatic method using esterase and cholesterol oxidase, and glucose levels were determined by enzymatic method with hexokinase (Cobas Integra 400 plus Roche, Switzerland). The total recovery of all the steps of extracting and processing the samples (mean ± SD) was 86 ± 4%.

### 2.5. Chemical Analyses

#### 2.5.1. Reagents

For the preparation of standard solutions, dilutions of the sample, and washing of the whole equipment and glass, double-distilled water (DDW) obtained from Barnstead, Easypure UV was used. For the validation of the ICP-AES measurement, working calibration solutions of the tested micro and macroelements (Zn, Ni, Fe, Mn, Cu, Mg, Na, K) (Merck KGaA, Darmstadt, Germany) were used.

#### 2.5.2. Instruments

The MDS-2000 microwave mineralization system (maximum power of 1000 watts, maximum pressure of 13.8 bar, and maximum operating temperature of 200 °C) was used in all digestion procedures of the tested red blood cells. Before analyzing the elements by ICP-AES, each 1.0 ± 0.1 mL sample was digested with 5 mL of nitric acid (69% Merck KGaA, Gernsheim, Germany) and 2 mL of H_2_O_2_ (35% *w*/*v*). Three blank tests were carried out under the same conditions. All samples were stored at 4 °C in a refrigerator until analysis by ICP-AES (inductively coupled plasma-atomic emission spectrometry).

Nominal values of the analyzed concentrations of micro and macroelements were determined using an ICP-AES apparatus (Yobin Yvon JY-24) equipped with the Meinhard TR 50-C1 nebulizer. The generator operated with an output power of 1000 W; frequency, 40.68 MHz; the plasma gas, the auxiliary gas, and the nebulizer gas were argon with flow rates of 12.0, 1.0, and 1.1 mL/min, respectively. The wavelengths (nm) used for the detection of Zn, Ni, Fe, Mn, Mg, Na, and K were 213.9, 231.6, 238.2, 257.6, 279.5, 589.5, and 766.5 respectively. The accuracy and precision of the methods used were tested with the certified reference material Trace Elements Whole Blood L-1 (Seronorm, Norway). The limit of quantification (LOQ) was performed on ultrapure blank reagents based on the usual LOQ criteria of 10 blind standard deviations [25]. The limits of detection (LOD) and LOQ of the ICP-AES methods ranged between 0.5–3.85 μg/g and 0.9–12.65 μg/g, respectively. The LOD and LOQ for the individual elements were (in µg /g): Zn (1.1; 3.33), Ni (0.5; 0.9), Fe (1.2; 3.96), Mn (0.6; 2.1), Cu (1.6; 4.1), Mg (2.2; 3.6), Na (3.85; 10.22), and K (3.33; 12.65), respectively.

### 2.6. Statistical Analysis

The results were statistically analyzed using the software package Statistica 13.0 (Statsoft, Cracow, Poland). The arithmetical mean, standard deviation, and the significance of differences were calculated using ANOVA. The normality of the data was checked using the Kolmogorov-Smirnov test. An independent t-test was used to compare two groups of women with PCOS (with and without insulin resistance). The differences in the content of selected biochemical parameters between PCOS women with and without insulin resistance were compared using Mann-Whitney U. Pearson’s correlation statistics were used to determine the correlation between the variables. Statistical analyses were performed at the significance level of α = 0.05. To control type I errors, the false discovery rate (FDR) approach was used. The calculations were performed using the p.adjust function of the stats package in R (R Foundation for Statistical Computing, Vienna, Austria, https://cran.r-project.org (accessed on 5 May 2021)).

## 3. Results

The statistical analysis showed significant differences between the anthropometric parameters (Table 1) as well as biochemical parameters (Table 2). Table 2 presents the concentrations of the following measured parameters: insulin, glucose, HOMA-IR, LDL, HDL, cholesterol, and triglycerides.

The main result of the research was the detection of the relationship between the level of trace elements in erythrocytes (Table 4) and the glycemic markers and lipid parameters of women with PCOS. Based on HOMA-IR ≥ 2.5, insulin resistance was found in 28 patients with PCOS (60%), while 19 patients (40%) had no insulin resistance. The comparison of the trace element content in erythrocytes in PCOS patients with and without insulin resistance is presented in Table 2. Despite the anthropometric and biochemical differences between PCOS women with insulin resistance and without it, the levels of the elements did not differ (Table 2). The adjustment for confounding factors confirmed the observed relationships.

When examining lipid and glycemic markers in women with PCOS, no statistically significant differences were found between the studied Fe, Mn, Cu, and K in both of the subgroups mentioned. We also found no difference in glycemic markers between women with PCOS with and without insulin resistance.

The results of PCOS studies in women without insulin resistance indicated significant relationships (*p* < 0.05) between HDL and several elements (Table 5). As the HDL level increased, the amount of zinc and magnesium increased, while the amount of nickel and sodium decreased. In women with insulin resistance, we noticed that nickel levels in RBCs increased with increasing LDL levels (*p* < 0.05).

## 4. Discussion

In recent years, research has focused on the relationship between micro and macroelements and human health. Benaglia et al. [26] found that many gynecological diseases, incl. infertility, recurrent miscarriages, and tumors are associated with abnormal metabolism of elements. So far, data on mineral levels in women with PCOS have not been adequately analyzed. There are only a limited number of scientific publications in the literature on the levels of micro and macroelements in blood cells. Most of the research concerns the minerals in the blood plasma, which does not seem to be the right approach. Zinc, manganese, and copper are essential micronutrients contained in many metalloenzymes and proteins responsible for cellular metabolism and the pathways regulating oxidative stress [27]. We assumed that there may be a link between these trace elements and oxidative stress-related PCOS.

Zinc may be associated with the development of polycystic ovary syndrome and its long-term metabolic complications through participation in glucose metabolism and the synthesis, secretion, and signaling of insulin [18]. In addition, zinc is a stabilizer of the insulin complex in beta-cell secretory granules [28]. Although studies by many authors indicate that women with PCOS have low levels of zinc compared to healthy people, it is unclear whether this is caused by improper intake or absorption, increased excretion, or the need for zinc [5,27,29]. Some indicate that zinc deficiency may lead to a decrease in antioxidant capacity, causing insulin resistance and apoptosis [29,30]. It is also observed that in the case of zinc deficiency, insulin is less stable and degrades faster [28]. The reference range of zinc in red blood cells is 8–14.5 (µg/g). In our study, the zinc content in red blood cells of women with PCOS ranged from 7.2 to 16.6 mg /l (10.0 ± 1.9 mg/L) and did not differ significantly from the level in healthy women. Being aware that Zn stabilizes insulin hexamers and participates in its storage in the pancreas, we suspected that in women with insulin resistance there would be much less of it. However, our results did not show a significant relationship between glycemic markers and the concentration of this element in the erythrocytes of women with PCOS. Only in the case of PCOS-NIR, zinc deficiency decreased the HDL level in a statistically significant way. Several human studies have shown that zinc supplementation lowers total cholesterol, LDL, and triglycerides, and increases HDL levels [31,32,33]. In our study, we also observed a significant positive correlation between the amount of zinc in erythrocytes and the level of HDL, but only for PCOS-NIR women. However, other studies suggest that the lipid profile is not zinc dependent [34,35,36,37]; therefore, the division of the study group into PCOS-IR and PCOS-NIR may explain these discrepancies.

Copper is an important element in the formation of red blood cells, bones, and connective tissue. About 80 percent of the copper in erythrocytes is bound to the enzyme superoxide dismutase. It is suspected that PCOS may be involved in the dysregulation of systemic copper homeostasis [18,38]. Prodarchuk et al. [39] found a tendency to increase serum copper concentration in women with PCOS, but the differences were not confirmed statistically. However, literature data indicate that there is a relationship between elevated copper levels in the body and PCOS [39,40,41]. Copper ions are believed to act as a catalyst in the synthesis of highly reactive oxygen species (ROS), leading to oxidative stress [41]. It has also been shown that long-term exposure to Cu^2+^ can damage the overall function of the ovaries [16]. Copper combined with homocysteine in the form of Cu-Hcy complexes increases the risk of contracting early vascular diseases in women with PCOS [39]. The reference range for copper in red blood cells is 0.52–0.89 (µg/g). In our study, they ranged from 0.48 to 0.98 µg/g. Although studies indicate that serum copper levels are significantly higher in obese patients compared with the normal-weight control group, we did not find such an association in our study (a study in erythrocytes). Some authors report a negative correlation between serum copper and cholesterol and HDL [4], but in this case no correlation was observed.

Despite the inclusion of nickel by WHO in the group of potentially essential micronutrients, the literature data on its role in regulating reproductive functions is practically absent. It is known, however, that nickel is capable of inducing the oxidation of free radicals in cell membranes and may participate in damaging folliculogenesis and ovulation in patients with PCOS [9]. In this study, we found a slight decrease in nickel content in patients with PCOS (both groups), which, however, due to the lack of available literature, does not apply to other authors. However, analyzing the level of nickel in the serum of women with PCOS indicated an increased level of this element in PCOS patients compared with the control group [27,38,42,43]. The authors of the study suggested that the increased level of Ni exposure in the blood serum may be the cause or background factor of PCOS [40]. In our study, we observed two significant correlations between nickel and the lipid profile of PCOS patients, namely in women with insulin resistance; as the amount of nickel increased, the level of LDL increased, but in patients without insulin resistance, the level of LDL HDL decreased. This effect is difficult to explain, but confirms the observation of Prodarchuk et al. [39] that nickel levels may have a negative impact on the fluid profile of PCOS patients.

Of the iron in the body, 80% binds to hemoglobin. At low levels of this element, iron-dependent hemoglobin is not synthesized in sufficient quantities, and the oxygen-carrying capacity of red blood cells is limited, which in turn leads to anemia [44,45]. Studies by many authors have shown that the level of iron in the serum and body of PCOS patients is elevated and is a probable cause of coronary artery disease [46,47,48]. In our study, iron ranged from 559.2 mg/L to 1333.4 mg/L (reference iron concentration in erythrocytes: 745–1050 mg/L) and was slightly elevated in people with insulin resistance, but these differences were not statistically significant. No statistically significant differences were found compared to the control group; therefore, it seems that iron excess did not play a significant role in the development of coronary artery disease in the studied group with PCOS. It proves a better state of this element resulting from higher meat consumption.

Manganese is a trace element that causes oxidative stress by disrupting glucose metabolism [7,17]. Some studies have found that manganese may be involved in a disorder of glucose metabolism in women with PCOS. Kurdoglu et al. [16] observed that serum manganese levels were decreased in women with PCOS. In our study of erythrocytes, we also found slightly less manganese in women with PCOS than in the control group, but it was not statistically significant. Some researchers, however, observed increased levels of this element in the PCOS group compared with the control group [42,49]. These authors suggest that the increased level of manganese exposure can be attributed to PCOS factors or cofactors [43]. These differences may be due to the material studied (serum, erythrocytes), but since no differences were found in our own study, it seems that manganese plays a relatively minor role in abnormal glycemic markers in women with PCOS.

Magnesium is closely associated with type-2 diabetes and other metabolic diseases. This element plays an essential function in glucose metabolism, acting as a cofactor for many enzymes, as well as being part of the Mg^2 +^ -ATP complex [50]. Mg also affects the activity of insulin [51]. In our study, decreased membrane fluidity of RBC was associated with higher levels of plasma insulin. It is believed that magnesium deficiency can cause oxidative stress [19,45]. Hypomagnesaemia reduces the sensitivity of peripheral tissues to insulin by diminished autophosphorylation of tyrosine kinase, a component of the insulin β subunit of which magnesium is a cofactor [50]. Hypomagnesaemia may also be associated with decreased β-cell proliferation, thus affecting insulin production [51,52]. The magnesium content of red blood cells is a very good indicator of short-term magnesium status, and a low level indicates nutritional deficiencies [19]. In the presented study, the magnesium content in erythrocytes ranged from 34.0–66.8 mg/L and was slightly higher in women with PCOS than in the control group (statistically insignificant), whereas Mhaibes et al. [49] and Swetha et al. [53] observed significantly lower concentrations of this element in the serum of PCOS patients compared with the control group. There are also studies in which no such relationship was found [38,54,55]. The influence of magnesium levels on pathology of PCOS remains unclear [5]. It has been suggested that too little magnesium, whether caused by a poor diet, lifestyle, or other factors, may prevent the transport of glucose into cells in adequate amounts. As a result, people with insulin resistance tend to feel fatigue and difficulty controlling blood sugar levels. Suitable levels of magnesium may reduce insulin resistance and therefore reduce the risk of type-2 diabetes [55]. Mhaibes et al. [49] observed significantly lower concentrations of this element in the serum of PCOS patients compared with the control group. Our research may confirm Swetha et al. [53], who studied magnesium in red blood cells. Little is known about the ability of cellular proteins to bind Mg^2 +^. The cell membrane without the stabilizing effect of the magnesium complexation with phospholipids has an increased permeability, which leads to excessive accumulation of sodium and a decrease in the concentration of potassium in the cell. This is confirmed by our observations of the potassium content in red blood cells in PCOS patients. In cells lacking intracellular compartments, such as erythrocytes, Mg^2+^ buffering depends mainly on ATP, phosphonucleotides, and phosphometabolites, proteins and metabolic pools [56]. Mg increased HDL in PCOS-NIR statistically significantly; unfortunately, no satisfactory explanation of this relationship was found in the available literature.

Na, K-ATPase is an ion transport protein across the membrane, the activity of which determines and maintains high concentrations of K^+^ and low Na^+^ in the cytoplasm, generates potential across the membrane, and provides a driving force for secondary ion transport [57]. Ionic homeostasis maintained by the Na, K-ATPase is essential for many cellular functions and processes, including height, differentiation, migration, contraction, secretion, and regulation of cell volume [58]. The imbalance, by increasing the sodium concentration in the intercellular spaces, can contribute to water retention, i.e., dehydration of the cells, and thus disrupt gradient-based transport. Hyperinsulinemia plays an important role in the development of hypertension by increasing sodium retention, thereby leading to an increase in intracellular sodium concentration [56]. The average specific activity of Na, K -ATPase in healthy people is higher than among people with diabetes in the same age and the same gender, which may be associated with insulin levels. If insulin levels are low, the rate of glycolysis in red blood cells slows down, leading to a reduction in ATP levels and thus a reduction in enzyme activity. It was found that, in the case of in vitro treatment of erythrocytes in diabetic patients, insulin stimulates the increase in its activity, but did not restore it to normal [57]. Although in our study we did not find significant differences in the concentration of sodium or potassium, we can observe a slightly higher, statistically insignificant increase in potassium in women with PCOS (both with IR and without IR) compared with CG. The level of potassium in the red blood cells is the best single measure of the level of potassium in the body. Potassium depletion in the body can lead to a wide variety of effects, including high blood pressure, abnormal heart rhythms, and muscle weakness.

Incubation of human RBCs with glucose at a higher concentration significantly lowered Na, K-ATPase activity and increased lipid peroxidation and glycosylation of RBC membrane proteins compared with glucose-treated normal RBCs [59,60,61]. In adult patients with type-2 hypercholesterolaemia, in addition to changes in the structure and fluidity of erythrocyte membranes, impairment of the function and structure of plasma membrane proteins has been observed. This has been documented by a reduction in the concentration of thiol groups in membrane proteins, followed by a lower Na, K-ATPase activity. Testosterone mediates this effect on vascular reactivity by blocking the calcium channel (L-calcium channel) and stimulating the potassium channel opening. Due to the higher levels of free testosterone in women with PCOS, it seems obvious that the flow through the canal is impaired, including the metabolism of magnesium, sodium, and potassium. These disorders contribute to the dysregulation of glucose metabolism [61].

## 5. Conclusions

The study found that women with PCOS had lower amounts of Zn, Ni, Mn, and Cu in their erythrocytes compared with healthy women. It seems that a rich diet or supplementation with minerals in the PCOS group is required. It is worth considering introducing products rich in these elements into the diet. The increased levels of macroelements (Mg and K) found in PCOS women due to impairment of Na, K-ATPase activity and the L-calcium channel appeared to have a positive protective effect. Unfortunately, an increased content of Na in red blood cells was also found, which negatively affects the body, including by retaining water in the body. In the PCOS-NIR group, higher Mg and Zn contents favored higher HDL levels and higher Na and Ni levels lower HDL levels; the relationship is difficult to explain and, therefore, more research is needed.

## Figures and Tables

**Table 1:** Anthropometric characteristics of the study group.

Parameters	PCOS-IR (n = 28)		PCOS-NIR (n = 19)		TOTAL PCOS (n = 47)		CG (n = 16)	
	Mean	SD	Mean	SD	Mean	SD		
Age (y)	27.7	5.5	29.2	3.4	28.3	4.6	29.0	4.4
Height (m)	1.67 ^a^*	0.06	1.67 ^a^	0.05	1.67	0.06	1.68	0.05
body weight (kg)	90.13 ^a^	16.08	72.28 ^b^	10.24	82.91	16.47	65.8	6.2
BM (kcal)	1536 ^a^	177	1426 ^b^	97	1492	158	1404	96
TM (kcal)	2181 ^a^	239	2053 ^b^	141	2129	213	2066	122
Na/K	0.93 ^a^	0.19	0.99 ^a^	0.13	0.95	0.17	1.19	0.82
BCMI	10.17 ^a^	2.99	9.05 ^a^	2.59	9.72	2.86	9.8	2.81
TBW (L)	38.12 ^a^	4.88	33.74 ^b^	3.24	36.35	4.78	31.92	4.07
TBW (%)	42.78 ^a^	4.29	46.54 ^b^	5.03	44.30	4.92	50.8	5.22
TBW IN (L)	20.23 ^a^	4.25	17.33 ^b^	2.39	19.06	3.86	16.88	2.51
TBW IN (%)	52.89 ^a^	6.95	51.27 ^a^	3.76	52.23	5.88	51.78	4.86
TBW EX (L)	17.86 ^a^	3.23	16.41 ^a^	1.77	17.27	2.80	15.3	2.81
TBW EX (%)	46.96 ^a^	7.07	47.09 ^a^	8.22	47.01	7.47	47.0	7.14
Phase angle (PA)	6.87 ^a^	5.42	5.44 ^a^	0.73	6.29	4.24	6.38	4.83
fat mass (kg)	38.31 ^a^	11.64	26.82 ^b^	9.05	33.67	12.00	20.12	7.48
fat mass (%)	41.85 ^a^	7.49	36.55 ^a^	8.74	39.71	8.35	30.9	8.69
BCM (kg)	27.39 ^a^	7.51	23.34 ^b^	3.34	25.75	6.45	22.7	5.14
BCM (%)	51.40 ^a^	8.78	50.53 ^a^	4.01	51.05	7.19	50.31	4.17
muscle m (kg)	36.45 ^a^	8.90	30.91 ^b^	6.83	34.21	8.50	30.26	6.96
muscle m (%)	41.34 ^a^	12.02	43.11 ^a^	9.75	42.05	11.08	48.44	11.64
BMI (m^2^/kg)	32.64 ^a^	6.26	26.00 ^b^	3.41	29.95	6.20	2.3	3.8
WHR	0.96 ^a^	0.07	0.87 ^b^	0.06	0.93	0.08	0.79	0.03

PCOS-NIR—PCOS with no -insulin resistance; PCOS-IR—PCOS with insulin resistance; a, a*, b letters—significant differences between PCOS-IR and PCOS-NIR. BM—basic metabolism; TM-total metabolism; BCMI—body-cell-mass index; TBW—total body water; TBW IN—total intracellular water TBW EX—total extracellular water; BCM—body-cell mass; BMI—body mass index; WHR—waist-hip ratio.

**Table 2:** Biochemical differences between PCOS with and without insulin resistance.

Parameters	PCOS-IR (*n* = 28)		PCOS-NIR (*n* = 19)		TOTAL (*n* = 47)	
	Mean	SD	Mean	SD	Mean	SD
DHEA-SO_4_ (μg/d)	246.81	90.00	236.89	89.66	242.80	89.02
Androstendione (ng/mL)	3.54	1.05	3.84	1.63	3.66	1.31
Estradiol (ng/mL)	43.76	11.53	35.80	14.31	40.54	13.18
SHBG (nmol/L)	32.59	13.08	44.07	20.77	37.23	17.37
Testosterone (ng/mL)	1.04	1.39	0.48	0.19	0.81	1.11
Insulin test 0 (mU/L)	15.79 ^a^*	4.29	7.11 ^b^	1.93	12.28	5.55
Insulin test after 2 h (mU/L)	99.12 ^a^	50.02	45.02 ^b^	27.14	77.25	49.77
Glukose test 0 (mg/dl)	94.07 ^a^	9.01	88.45 ^b^	9.09	91.80	9.36
Glukose test after 2 h (mg/dL)	123.9 ^a^	30.4	100.0 ^b^	19.6	114.24	28.87
IR	0.17 ^a^	0.05	0.08 ^b^	0.02	0.13	0.06
HOMA-IR	3.66 ^a^	1.02	1.56 ^b^	0.47	2.81	1.33
Total cholesterol (mg/dL)	185.4 ^a^	27.9	184.8 ^a^	24.6	185.2	26.3
LDL (mg/dL)	120.8 ^a^	27.3	104.2 ^b^	20.1	114.1	25.7
TG (mg/dL)	123.3 ^a^	55.2	95.3 ^a^	41.0	112.0	51.4
HDL (mg/dL)	51.0 ^a^	12.3	65.8 ^b^	17.5	57.0	16.2

PCOS-NIR—PCOS with no insulin resistance; PCOS-IR—PCOS with insulin resistance; a, a*, b letters—significant differences between PCOS-IR and PCOS-NIR. DHEA-SO_4_—dehydroepiandrosterone sulfate, SHBG—sex hormone-binding globulin; IR—insulin resistance; HOMA—homeostasis model assessment; LDL—low-density lipoprotein; TG—triglyceride; HDL—high-density lipoprotein.

**Table 3:** Trace element content in diets (µg/g).

Element	PCOS-IR *n* = 28		PCOS-NIR *n* = 19		Control Group (CG) *n* = 16		*p*-Value * PCOS-IR vs. PCOS-NIR	*p*-Value * PCOS-IR vs. CG	*p*-Value * PCOS-NIR vs. CG
	Mean	SD	Mean	SD	Mean	SD			
	(Range)		(Range)		(Range)				
Zn	9.40	2.32	8.20	1.95	8.96	2.28	0.381	0.871	0.711
	(5.30–14.79)		(6.18–12.62)		(5.20–12.60)				
Fe	12.03	4.86	11.12	4.20	11.22	4.04	0.869	0.888	0.746
	(6.47–23.30)		(7.76–21.04)		(5.13–16.07)				
Cu	1.19	0.49	1.07	0.35	1.08	0.41	0.791	0.788	0.843
	(0.55–2.43)		(0.70–1.91)		(0.48–1.80)				
Mg	282.21	88.54	328.13	85.60	299.01	55.85	0.336	0.852	0.682
	(176.77–583.99)		(204.38–472.19)		(201.50–385.15)				
Na	3024.99	844.38	2542.22	770.89	2867.27	468.18	0.245	0.847	0.572
	(2066.85–5073.79)		(1329.71–3795.77)		(2001.1–3598.2)				
K	3704.23	2124.93	3157.57	1165.88	3551.73	379.06	0.659	0.966	0.830
	(1559.0–8430.1)		(1967.2–5362.3)		(3006.2–4012.5)				

PCOS-NIR—PCOS with no insulin resistance; PCOS-IR—PCOS with insulin resistance; * no significant differences were found between PCOS-IR and PCOS-NIR and the control group (*p* > 0.05).

**Table 4:** Average content of elements in erythrocytes (µg/g).

Element	PCOS-IR *n* = 28		PCOS-NIR *n* = 19		Control Group (CG) *n* = 16		*p*-Value * PCOS-IR vs. PCOS-NIR	*p*-Value * PCOS-IR vs. CG	*p*-Value * PCOS-NIR vs. CG
	Mean	SD	Mean	SD	Mean	SD			
Zn (µg/g)	10.14	2.11	9.89	1.44	10.30	1.67	0.910	0.970	0.825
Ni (µg/g)	0.001	0.0009	0.001	0.0006	0.002	0.000	0.348	0.230	0.116
Fe (µg/g)	868.0	155.8	835.3	156.4	833.0	94.6	0.766	0.799	0.999
Mn (µg/g)	0.017	0.006	0.017	0.008	0.020	0.009	0.970	0.527	0.653
Cu (µg/g)	0.714	0.129	0.713	0.114	0.761	0.146	0.999	0.608	0.594
Mg (µg/g)	48.4	8.3	50.0	8.4	45.3	10.7	0.849	0.637	0.359
Na (µg/g)	374.3	84.3	396.3	66.6	367.9	88.9	0.678	0.975	0.620
K (µg/g)	2541.8	330.9	2409.6	347.1	2336.9	211.4	0.401	0.203	0.813

PCOS-NIR—PCOS without insulin resistance; PCOS-IR—PCOS with insulin resistance; * no significant differences were found between PCOS-IR and PCOS-NIR and the control group (*p* > 0.05).

**Table 5:** Correlations of mineral composition with parameters of glycemic markers and lipid metabolism, taking into account the presence of insulin resistance.

	Zn (µg/g)	Ni (µg/g)	Fe (µg/g)	Mn (µg/g)	Cu (µg/g)	Mg (µg/g)	Na (µg/g)	K (µg/g)
Insulin test 0 (mU/L)	PCOS-NIR	PCOS-NIR	PCOS-NIR	PCOS-NIR	PCOS-NIR	PCOS-NIR	PCOS-NIR	PCOS-NIR
	*p* = −0.270	*p* = 0.22	*p* = 0.050	*p* = −0.091	*p* = −0.197	*p* = −0.258	*p* = 0.148	*p* = −0.290
	PCOS-IR	PCOS-IR	PCOS-IR	PCOS-IR	PCOS-IR	PCOS-IR	PCOS-IR	PCOS-IR
	*p* = −0.003	*p* = 0.112	*p* = 0.193	*p* = −0.063	*p* = 0.108	*p* = −0.218	*p* = −0.177	*p* = 0.133
Insulin test after 2 h (mU/L)	PCOS-NIR	PCOS-NIR	PCOS-NIR	PCOS-NIR	PCOS-NIR	PCOS-NIR	PCOS-NIR	PCOS-NIR
	*p* = −0336	*p* = 0.010	*p* = −0.069	*p* = −0.027	*p* = −0.336	*p* = −0.190	*p* = 0.207	*p* = −0.006
	PCOS-IR	PCOS-IR	PCOS-IR	PCOS-IR	PCOS-IR	PCOS-IR	PCOS-IR	PCOS-IR
	*p* = 0.044	*p* = 0.058	*p* = −0.005	*p* = −0.011	*p* = 0.278	*p* = −0.164	*p* = 0.279	*p* = 0.192
Glukose test 0 (mg/dL)	PCOS-NIR	PCOS-NIR	PCOS-NIR	PCOS-NIR	PCOS-NIR	PCOS-NIR	PCOS-NIR	PCOS-NIR
	*p* = −0.396	*p* = 0.245	*p* = 0.121	*p* = 0.171	*p* = −0.158	*p* = −0.165	*p* = 0.143	*p* = −0.407
	PCOS-IR	PCOS-IR	PCOS-IR	PCOS-IR	PCOS-IR	PCOS-IR	PCOS-IR	PCOS-IR
	*p* = 0.304	*p* = −0.300	*p* = 0.275	*p* = 0.254	*p* = −0.062	*p* = 0.192	*p* = −0.018	*p* = 0.015
Glukose test after 2 h (mg/dL)	PCOS-NIR	PCOS-NIR	PCOS-NIR	PCOS-NIR	PCOS-NIR	PCOS-NIR	PCOS-NIR	PCOS-NIR
	*p* = −0.325	*p* = −0.026	*p* = −0.254	*p* = 0.058	*p* = −0.105	*p* = −0.200	*p* = 0.160	*p* = −0.003
	PCOS-IR	PCOS-IR	PCOS-IR	PCOS-IR	PCOS-IR	PCOS-IR	PCOS-IR	PCOS-IR
	*p* = 0.127	*p* = −0.216	*p* = 0.058	*p* = 0.087	*p* = −0.055	*p* = −0.063	*p* = 0.092	*p* = −0.063
Total cholesterol (mg/dL)	PCOS-NIR	PCOS-NIR	PCOS-NIR	PCOS-NIR	PCOS-NIR	PCOS-NIR	PCOS-NIR	PCOS-NIR
	*p* = 0.150	*p* = −0.132	*p* = 0.113	*p* = 0.102	*p* = −0.043	*p* = 0.439	*p* = −0.233	*p* = −0.150
	PCOS-IR	PCOS-IR	PCOS-IR	PCOS-IR	PCOS-IR	PCOS-IR	PCOS-IR	PCOS-IR
	*p* = 0.146	*p* = 0.360	*p* = −0.068	*p* = −0.094	*p* = 0.050	*p* = 0.094	*p* = −0.089	*p* = 0.017
LDL (mg/dL)	PCOS-NIR	PCOS-NIR	PCOS-NIR	PCOS-NIR	PCOS-NIR	PCOS-NIR	PCOS-NIR	PCOS-NIR
	*p* = −0.029	*p* = −0.074	*p* = 0.082	*p* = 0.234	*p* = −0.177	*p* = 0.250	*p* = −0.069	*p* = −0.210
	PCOS-IR	**PCOS-IR**	PCOS-IR	PCOS-IR	PCOS-IR	PCOS-IR	PCOS-IR	PCOS-IR
	*p* = 0.034	***p* = 0.501**	*p* = 0.113	*p* = 0.027	*p* = −0.080	*p* = 0.061	*p* = −0.122	*p* = −0.093
TG (mg/dL)	PCOS-NIR	PCOS-NIR	PCOS-NIR	PCOS-NIR	PCOS-NIR	PCOS-NIR	PCOS-NIR	PCOS-NIR
	*p* = −0.215	*p* = 0.213	*p* = −0.172	*p* = 0.010	*p* = 0.005	*p* = −0.139	*p* = 0.123	*p* = −0.337
	PCOS-IR	PCOS-IR	PCOS-IR	PCOS-IR	PCOS-IR	PCOS-IR	PCOS-IR	PCOS-IR
	*p* = 0.079	*p* = 0.251	*p* = −0.065	*p* = 0.006	*p* = 0.146	*p* = 0.063	*p* = 0.084	*p* = 0.093
HDL (mg/dL)	**PCOS-NIR**	**PCOS-NIR**	PCOS-NIR	PCOS-NIR	PCOS-NIR	**PCOS-NIR**	**PCOS-NIR**	PCOS-NIR
	***p* = 0.611**	***p* = −0.509**	*p* = 0.259	*p* = −0.257	*p* = 0.207	***p* = 0.519**	***p* = −0.559**	*p* = 0.238
	PCOS-IR	PCOS-IR	PCOS-IR	PCOS-IR	PCOS-IR	PCOS-IR	PCOS-IR	PCOS-IR
	*p* = 0.128	*p* = −0.017	*p* = −0.024	*p* = 0.023	*p* = −0.135	*p* = −0.077	*p* = 0.257	*p* = 0.125

PCOS-NIR—PCOS with no insulin resistance; PCOS-IR—PCOS with insulin resistance; bold—significant differences *p* < 0.05; LDL—low-density lipoprotein; TG—triglyceride; HDL—high-density lipoprotein.

## Data Availability

Data available on request.
